# Development of the DANish Cognitive Screen for Cardiac Arrest Survivors (DANcSCA)

**DOI:** 10.1186/s40359-025-02648-6

**Published:** 2025-04-10

**Authors:** Anders Wieghorst, Vicky L. Joshi, Lars Evald, Tonny E. Andersen, Britt Borregaard, Jørgen Feldbæk Nielsen, Morten Tjørnlund, Ann-Dorthe Zwisler

**Affiliations:** 1 REHPA, The Danish Knowledge Centre for Rehabilitation and Palliative Care, Odense University Hospital, Nyborg, Denmark; Department of Clinical Research University of Southern Denmark, Odense, Denmark, Nyborg, Denmark; 2 Department of Psychology, University of Southern Denmark, Odense, Denmark; 3 Department of Cardiology, Odense University Hospital, Odense, Denmark; 4 Department of Clinical Research, University of Southern Denmark, Odense, Denmark; 5 Hammel Neurorehabilitation Centre and University Research Clinic, Hammel, Denmark; 6 University of Aarhus, Aarhus, Denmark; 7 Centre for Rehabilitation of Brain Injury, Copenhagen University, Copenhagen, Denmark; 8 Department of Physiotherapy and Paramedicine, School of Health and Life Sciences, Glasgow Caledonian University, Glasgow, UK; 9 Clinic of Palliative Medicine and Rehabilitation, Rigshospitalet, Copenhagen, Denmark; 10 Institute of Clinical Medicine, University of Copenhagen, Copenhagen, Denmark

**Keywords:** OHCA, Out-of-Hospital cardiac arrest, Cardiac arrest, Cognitive screening, Digital cognitive test, DANcSCA

## Abstract

**Background:**

Cognitive deficits are prevalent among cardiac arrest survivors, yet there is no consensus on standardised screening methods. Hence, a novel screening tool is needed to assist healthcare professionals in determining the necessity of further assessment or rehabilitation.

This paper reports on the development process of a cognitive screening for cardiac arrest survivors and makes recommendations for timing and how to communicate the results.

**Methods:**

Based on an iterative process that included clinical observations and roundtable discussions, we underwent a four-phase development process grounded in the GUIDED guidelines (Duncan E, BMJ Open 10:e033516, 2020).

**Results:**

During the first phase, we explored the cognitive after-effects of cardiac arrest, focusing on potentially affected cognitive functions, suitable tests, and cognitive rehabilitation needs. The second phase included developing a pen-and-paper neuropsychological screening battery, which proved too resource-intensive due to its reliance on neuropsychologists. Consequently, in the third phase, we transitioned to a tablet-based screening tool. The fourth phase involved proof of concept, assessing whether we had developed a feasible cognitive screening for cardiac arrest survivors that could be implemented in hospital departments and rehabilitation settings. For this procedure, we determined that the appropriate time for screening is six to eight weeks after hospital discharge, with screening results categorised as: 'no concerns,' 'need for further assessment,' and 'need for specialised rehabilitation. The screening, appropriate timing for its administration, and communication of results are presented.

**Conclusion:**

We have successfully developed and reported a digital screening for cognitive deficits following cardiac arrest. This approach has the potential to enable systematic screening of all cardiac arrest survivors.

**Supplementary Information:**

The online version contains supplementary material available at 10.1186/s40359-025-02648-6.

## Introduction

As the survival rate of cardiac arrest (CA) increases [1], so does the number of survivors with cognitive deficits. This growing population creates an increasing need for a valid and feasible clinical screening to detect their specific cognitive deficits.

Two systematic reviews by Moulaert et al. [2] and Zook et al. [3] document a highly heterogeneous approach to assessing cognitive functions and considerable variation in the criteria for defining cognitive deficits. However, both reviews confirm, based on high-quality studies, that 42–50% of CA survivors have cognitive deficits, most often with attention, memory, and executive function.

Even subtle cognitive deficits can influence the survivors’ quality of life, daily function, and return to work [4–6]. Thus, it is essential to identify survivors with cognitive deficits before returning to everyday life, social activities, and other cognitively demanding tasks. Equally, it is just as important to clear those with no cognitive deficits to reduce uncertainty for the survivors and their families in their future planning.

The European Guidelines 2021 on Post-resuscitation care [4] recommend screening for cognitive deficits and suggests a two-step approach: 1) simply asking survivors and relatives or using a structured questionnaire and 2) using the Montreal Cognitive Assessment (MoCA) tool and if there are signs of cognitive impairment consider referral to neuropsychologists for more extensive neuropsychological assessment [4]. However, simply asking survivors or relatives has been found to be inadequate since they may not always be fully aware of their cognitive deficits [5, 6]. Similarly, studies suggest that using the MoCA and its predefined cut-off score has limited predictive value in CA survivors [7, 8], and there is limited overlap between the cognitive domains suspected to be affected by CA and the cognitive domains included in the MoCA screening. While the MoCA is likely suited for identifying more severe cases of cognitive deficits, there is a risk that individuals may score enough points from unaffected domains to exceed the MoCA cut-off, despite having mild to moderate cognitive deficits. This risk is particularly pronounced in younger survivors and those with a high premorbid cognitive level, as they were likely further above the cut-off before the cardiac arrest, making it easier to meet the threshold despite deficits.

At present, the best practice for detecting these mild to moderate cognitive deficits is a thorough neuropsychological assessment [9–11]. However, this approach is very costly and time-consuming, often making it unfeasible in routine clinical practice. This underscores the need for a new screening that can efficiently identify even mild cognitive deficits in clinical settings without placing excessive demands on resources.

To date, there is no consensus on when to screen for cognitive deficits. Moulaert et al. [12] recommend early screening, preferably less than one month post-CA, while others have tested screening at different time points, from the acute phase till many years after [2, 3]. The results from Moulaert et al. (2015) [12] indicated that early detection of cognitive deficits after CA results in improved quality of life, better overall emotional state, reduced anxiety, and a quicker return to work. However, no standard procedures currently exist on how to communicate the screening results or their implications to survivors.

Our objective was to develop a cognitive screening tailored to the specific cognitive deficits characteristic of the cardiac arrest population for use in a clinical setting, including even mild yet clinically relevant deficits that are likely overlooked by the MoCA or other broader and coarser screening tools. This involved identifying the cognitive functions most affected, determining the appropriate timing for screening, and a clear communication of the results. This paper describes the development process in detail, enabling other researchers to build upon our work and avoid duplicating efforts, as shared experiences are key principles in development [13]. It includes considerations and choices but does not present statistical analyses, as those are reported in a separate validation article (unpublished).

## Methods

**Fig. 1:**
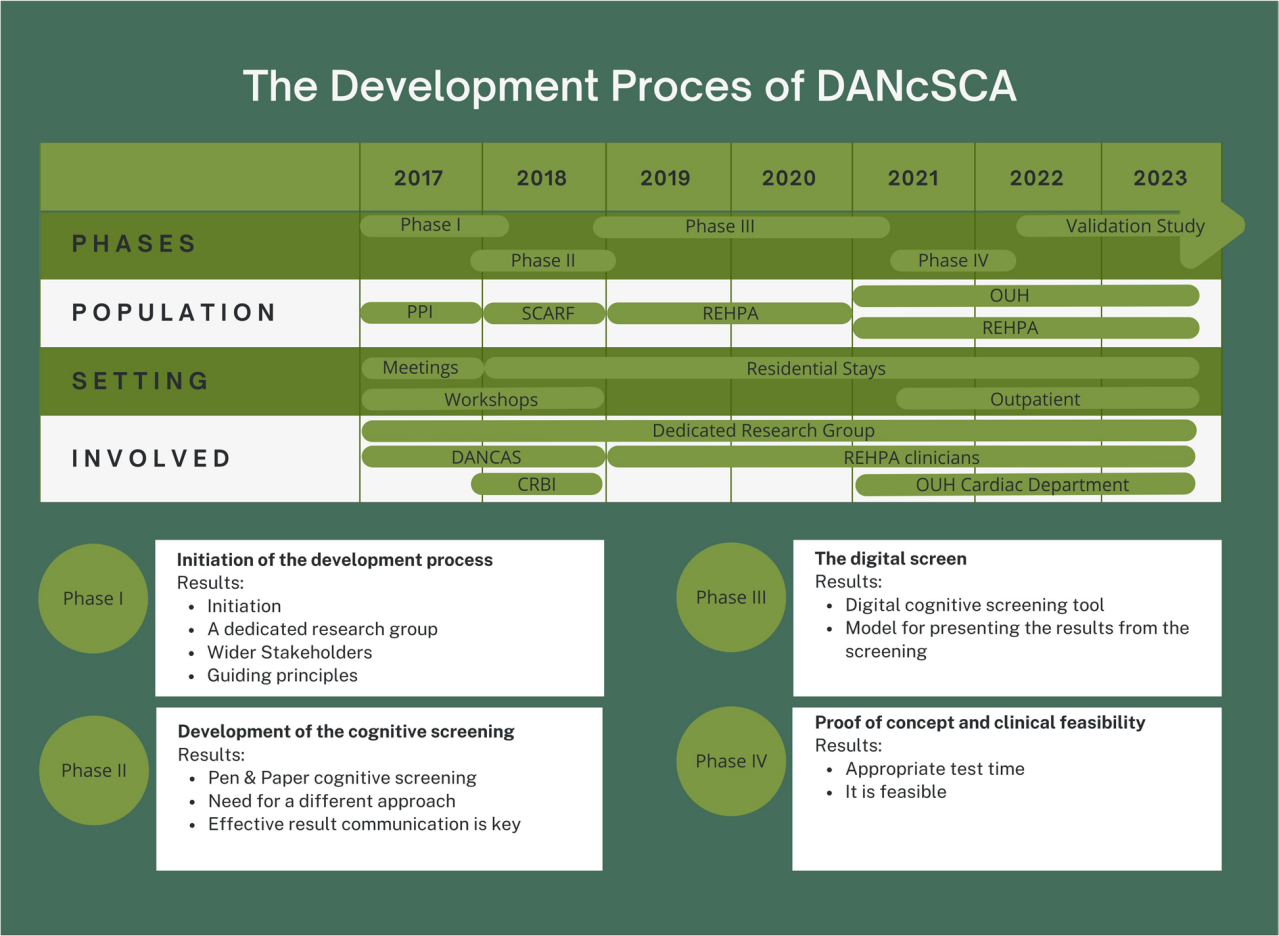
DANcSCA: Development of DANish Cognitive Screen for Cardiac Arrest Survivors PPI: Public patient involvement SCARF: The “Survivors of Cardiac ARest focused on Fatigue” study REHPA: The Danish Knowledge Centre for Rehabilitation and Palliative Care OUH: Odense University Hospital CRBI: Centre for Rehabilitation of Brain Injury, Copenhagen

**Fig. 2:**
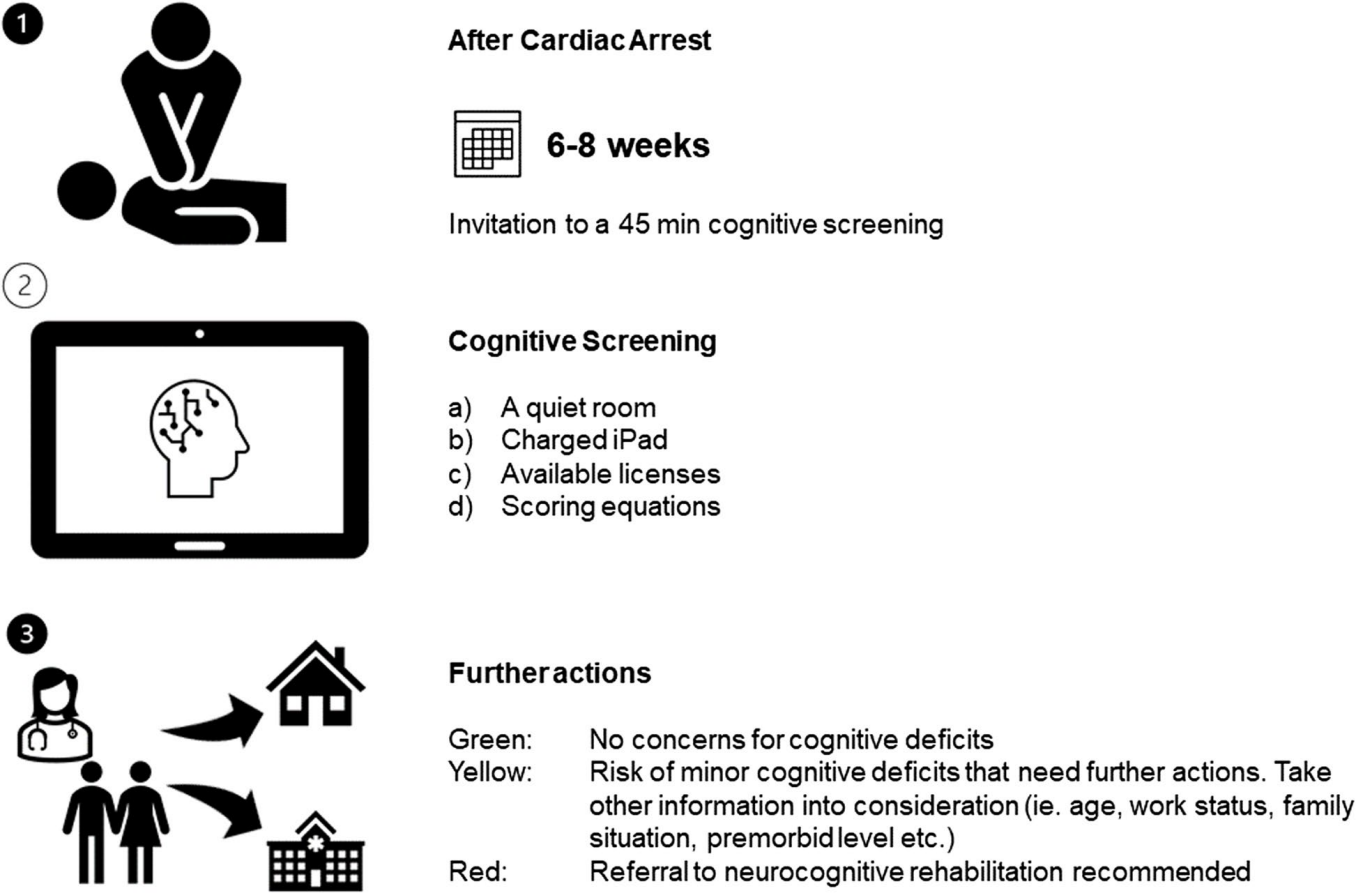
The final model of the screening procedure

Based on an iterative process that included roundtable discussions, clinical observations, and clinical testing (see Fig. 1), we developed the DANsCA cognitive screening (see Fig. 2) in collaboration with researchers, clinicians, patients, and their relatives.

The development process is reported in accordance with the GUIDance for the rEporting of intervention Development (GUIDED) guidelines [14].

A review of the existing literature and discussions from the first DANCAS roundtable meeting in 2017 highlighted a lack of consensus on the prevalence of cognitive deficits, the specific cognitive functions affected, and the methods for identifying survivors with these deficits. The key evidence came from two studies by Moulaert et al. [2, 12] and the clinical experience of the experts involved.

In response, we initiated a four-step development process grounded in the GUIDED guidelines [14]. The first phase was *Target Population-centered development* [14], focusing on identifying the cognitive functions most frequently affected in CA survivors and the extent of these deficits. Following this, the development approach evolved toward an *implementation-based approach* [14], which concentrated on adapting and refining the screening tool to enhance the likelihood of successful implementation in clinical settings. In the third phase, we transitioned to an *evidence and theory-based development approach* [14] as more studies had been published by this time [15–17]. The fourth and final phase involved assessing the proof of concept and the feasibility of utilizing the tablet-based screening tool in clinical settings—an essential step before moving to a validation study that assessed validity and determined the cut-off calculations (unpublished). This phase focused only on evaluating the clinical feasibility of using a tablet-based screening, while clinical decisions at this stage were still based on traditional neuropsychological tests.

### Target population and participants

The development process included input from two public patient involvement (PPI) groups and three patient cohorts, consisting of participants who were ≥ 18 years old, able to speak and understand Danish, and capable of completing a neuropsychological assessment. See Table 1 for a further description of the participants.

**Table 1:** Overview of participants in the development process as part of roundtable discussions, consensus meetings and clinical testing

Target population	Phases	Characteristics	Role	Inclusion criteria
PPI (DANCAS)	I, II, III & IV	2 CA survivors 1 Relative	Roundtable discussions and consensus meetings	CA survivor or relative
Workshops	II	24 CA survivors 15 Relatives	Clinical testing	Self-identified need for rehabilitation
SCARF	II	40 CA survivors 24 Relatives	Clinical testing	CA > 3 month Self-identified need for rehabilitation
REHPA	III	17 CA survivors 11 Relatives	Feasibility	CA > 3 month Self-identified need for rehabilitation
OUH	IV	17 CA survivors	Feasibility	CA 6 to 8 weeks prior

*PPI* Public Patient Involvement, *CA* cardiac arrest, *DANCAS* Danish Cardiac Arrest Survivorship, *SCARF* Survivors of Cardiac ARest focused on Fatigue study, *REHPA* The Danish Knowledge Centre for Rehabilitation and Palliative Care, *OUH* Odense University Hospital

### Setting

The DANcSCA was developed within the Danish universal and tax-funded healthcare system. There are approximately 800 new out-of-hospital CA survivors per year in Denmark [1]. If the cause of CA is ischemic heart disease, survivors are usually offered cardiac rehabilitation [18]. However, this provision is inconsistent across Denmark, and there is no targeted rehabilitation for CA survivors [18].

### Stakeholder contribution

A dedicated research group (three psychologists, a neurologist, a nurse, and a cardiologist) led the development process, guided by a broad range of stakeholders, including occupational therapists, physiotherapists, and social workers with expertise in post-CA treatment and rehabilitation.

The Danish Cardiac Arrest Survivorship (DANCAS) network, a national initiative uniting clinicians, researchers, survivors, and other stakeholders, contributed expertise on the CA population and the Danish healthcare system [19]. REHPA facilitated network meetings and the residential programme. Neuropsychological knowledge was provided by the Centre for Rehabilitation of Brain Injury (CRBI), the Department of Psychology at the University of Southern Denmark (SDU), and Hammel Neurorehabilitation Centre and University Research Clinic. Cardiac departments from Odense University Hospital (OUH) and Copenhagen University Hospital served as advisors.

### User-involvement

Three CA survivors, one relative of a CA survivor, and one representative from the Danish Heart Foundation participated continuously in the DANCAS workshops and were invited to contribute in accordance with the professional stakeholders. Furthermore, two user-involvement workshops were conducted as part of the development phase II, involving 24 CA survivors and 15 relatives who took part in testing sessions and interviews [19].

## Results

### Phase I: initiation of the development process

The first outcome of the development process was the formation of a dedicated research group and the involvement of broader stakeholders. Through collaborative work and discussions, three guiding principles for the screening process emerged:

1. Identify CA survivors who require referral for a comprehensive neuropsychological assessment.
2. Be easy to administer and interpret, including by non-psychologist healthcare professionals.
3. Produce results that are simple to communicate and understand.

### Phase II: development of the cognitive screening

The initial cognitive testing during the development process revealed deficits primarily in the cognitive domains of memory, working memory, attention, mental processing speed, and cognitive flexibility. These findings guided the focus of the initial version of the cognitive screening assessment. As further studies came to our attention [15–17] they confirmed the relevance of these domains.

We initially set aside guiding principle 2 (easy to administer and interpret) and utilized pen-and-paper neuropsychological tests that required the expertise of a neuropsychologist. At that time, we did not see a viable option to meet the principle without involving psychologists, as we continued to explore alternatives that could eventually be administered and interpreted by non-specialists.

The tool was used to test participants during their stays. It was evaluated by the Center for Rehabilitation of Brain Damage and the dedicated research group after each of the two user-involvement workshops, leading to the changes outlined in Table 2. Below, we highlight some of the more important rationales behind these changes, which were informed by clinical expertise rather than systematic data collection at that time.

**Table 2:** The versions of the pen-and-paper cognitive screening and cognitive domains

Cognitive domains	Test	1. version	2. version	3. version
Memory	Rey Complex Figure [20]	X		
	RBANS list learning [21]		X	X
Attention and speed	WAIS-IV Coding [22]	X	X	X
	d2 Test of Attention [23]	X		
	Trail A & B [24, 25]		X	X
Working memory	WAIS-IV digit span [22] Forward Backward	X	X	X
	NCCEA Sentence Repetition Test [26]	X	X	
	PASAT 2s [27]		X	X
Cognitive flexibility	Verbal Fluency Animals S-words [28]	X	X	X
	B/K alternating [29]	X	X	
	Five-point test [30]		X	X

The three versions of pen-and-paper cognitive screening for cardiac arrest survivors. All tests had to have reference data for all ages 20–90 years

We chose to exclude the Rey Complex Figure test for two reasons. First, it was unclear whether participants' recall problems were related to difficulties with strategy and organization when copying the figure. Moreover, administering the Rey Complex Figure test posed challenges in ensuring uniform test conditions (Principle 2).

For a better insight into executive functions, particularly cognitive flexibility, we added the Trail A and Trail B tests, which assess attention and processing speed. As a result, the d2 test was no longer necessary, and we excluded it, as the combination of WAIS-IV Coding and the Trail tests provided sufficient assessment of processing speed. Additionally, we included the Five-Point Test and PASAT in the test battery to further explore executive functions, as well as complex visual overview and planning.

We also observed an overlap between fluency with S-Words and fluency with Alternating Letters, making the inclusion of both tests redundant. Therefore, we decided to retain only fluency with S-Words, as it is more commonly used in research. Similarly, both the Sentence Repetition test and the WAIS-IV Digit Span assess working memory capacity, so we opted to retain the more frequently used WAIS-IV Digit Span.

### Results from Phase II

The evaluations revealed that the pen-and-paper cognitive screening was relevant and beneficial for CA survivors, their relatives, and health professionals. The screening provided insights that both survivors and their relatives could recognize and find meaningful while also offering useful information for tailoring rehabilitation initiatives. However, the resource-intensive nature of the pen-and-paper screening tool meant it did not align with our second guiding principle (easy to administer and interpret, including by non-psychologist healthcare professionals) leading us to adopt a different approach.

The presentation of results from the pen-and-paper screening was found to be overly complex. It required dedicated time to educate survivors about cognitive functions, their connection to CA, and how the deficits might be addressed. While this approach may be effective in a residential rehabilitation setting with ample time and resources, it does not meet our third guiding principle: Produce results that are simple to communicate and understand.

### Phase III: the digital screening approach

In order to meet guiding principles 2 (be easy to administer and interpret, including by non-psychologist healthcare professionals) and 3 (produce results that are simple to communicate and understand), we drew inspiration from the realm of cognitive research. Digital testing has become widespread within this realm due to its ease of administration, standardisation, reduced scoring errors, and immediate accessibility to tests and results without extensive material preparation [31]. Building on this concept, we developed an alternative approach: administering cognitive screening to CA survivors using a tablet. This approach involved a shorter test duration and potentional for immediate delivery of results. With this understanding, we proceeded to review the digital cognitive test market. Our review revealed a diverse market, with options ranging from small tests that appeared underdeveloped and under-researched, e.g. Willer, Pedersen [32], to more comprehensive batteries like the Cambridge Neuropsychological Test Automated Battery (CANTAB) [33]. We chose to compile the digital screening tool from subtests from the CANTAB as the system with the most sensitive sub-tests and with the greatest potential to be administered without assistance from healthcare professionals (Appendix A). We matched the affected cognitive domains identified through phases I and II with the described functions CANTAB assesses, leading to the screening tool described in Table 3.

**Table 3:** Description of the included sub-test from the CANTAB battery

Cognitive Domains	Test	Description
Memory	The Verbal Recognition Memory (VRM)	VRM assesses verbal memory and new learning. It measures the ability to encode and subsequently retrieve verbal information
	Paired Associates Learning (PAL)	Paired Associates Learning assesses visual memory and new learning
Attention and speed	Motor screening task (MOT),	Assessment of whether sensorimotor deficits or lack of comprehension will limit the collection of valid data
Working memory & executive function	Spatial Working Memory (SWM)	Requires retention and manipulation of visuospatial information, has notable executive function demands and provides a measure of strategy and working memory errors

All descriptions from cambridgecognition.com

### Results from Phase III

In addition to developing a digital cognitive screening, we explored various models for effectively communicating the results. The approach outlined in Phase II was not feasible in a clinical setting, as it was time-consuming and required specific settings and expertise that are not readily available in clinical practice. Through iterative testing and discussions with CA survivors, we refined our communication strategies. This process resulted in the stratification of results into three categories: 'no concerns,' 'need for further assessment,' and 'need for specialized rehabilitation'.

This evolution in our approach also led to a new way of thinking about screening. We adopted the belief that the primary goal when screening for cognitive deficits in CA survivors is to efficiently identify those with no risk while quickly referring those with significant cognitive deficits for further assessment and rehabilitation. This approach results in an intermediate group with mild to moderate deficits who are at risk of being overlooked by broader and coarser screening tools. In these cases, it is beneficial to consider additional clinical variables—such as work status, cognitive demands, and cognitive reserve—when deciding on further initiatives.

### Phase IV: proof of concept and clinical feasibility

This phase was conducted in an outpatient setting at OUH with a total of 17 CA survivors to assess two main aspects: a) the ability of CA survivors to complete the screening successfully concerning post-hospitalisation fatigue and medical conditions associated with CA, and b) the feasibility of utilising a tablet-based screening in clinical settings, including an evaluation of the spoken instructions delivered by the test.

### Results from Phase IV

Based on patients' responses and clinical findings, we found that testing six to eight weeks after the CA was an appropriate time. Although this is earlier than the recommended three months [4], most individuals were medically stable, had overcome post-CPR effects such as initial shock and ribcage pain, and had time to settle in at home after hospital discharge. Participants had not yet resumed work or other cognitively demanding tasks, allowing them the necessary time and energy for a cognitive screening.

Surprisingly, using the tablet was not an issue, even for those who had never used one. The stratification of the results into “no concerns,” “need for further assessment,” and “need for specialized rehabilitation” was easy to understand and effective for both clinicians and survivors, as well as their relatives. Only a few cases required further explanations, while the majority of participants expressed appreciation for the insights provided and the guidance on available options to navigate the new challenges.

Phase IV demonstrated that the DANcSCA screening is feasible in clinical settings. The app is available by CANTAB [34], though the cutoff scores and necessary equations for calculating the score are pending publication. As the screening was clinically feasible, the next step is to test its validity in a new study and preferably further refine the tool’s usability. These tasks fall outside the scope of this paper.

A proposed flow for the screening, inspired by the principles of the TIDieR framework [35], is presented in Fig. 2.

## Discussion

This paper reports on the development process of the DANcSCA. Through clinical testing, the DANcSCA has demonstrated significant potential in addressing the cognitive screening needs of CA survivors, as well as acceptable validity in a yet unpublished validation study. Through a four-phase approach, we created a screen that is not only feasible for use in clinical settings but also aligned with our guiding principles of easy administration, interpretation, and effective communication of results.

A key outcome of our approach was the adoption of a stratified screening process, classifying results into "no concerns," "need for further assessment," and "need for specialized rehabilitation." This new approach allows for more efficient identification of patients with either no risk, who can conclude their treatment, or significant cognitive deficits, who should be referred promptly. The intermediate group, however, should be managed within cardiac settings with the inclusion of other relevant clinical variables, such as anxiety, depression, and life circumstances. New interventions should be developed and tested specifically for this group.

Another key outcome is the implementation of a digital screening tool, which represents a shift from traditional cognitive screening methods. This tool allows non-psychologists to administer and interpret more sensitive tests compared to currently available screening tools, such as the MoCA.

An important finding from Moulaert, van Heugten [12] was that early detection of cognitive deficits and support in adjusting to the new situation can have positive benefits for CA survivors. However, Wagner et al. [36] found that screening too early may have poor associations with neuropsychological outcomes after three months. This may be due to confounding variables such as shock, fatigue, and the effects of hospitalization in the very early stages after CA.

In this study, testing at six to eight weeks after CA was identified as an appropriate time, as it seems to fall within a window between recovery from the pain and discomfort of CPR and before the resumption of cognitively demanding tasks, such as returning to work. These insights are based on unstructured observations and takeaways from informal discussions with CA survivors and should be studied further in future research.

The MoCA is widely used as a screening tool in the CA population and is suggested in guidelines, although it is not explicitly recommended [4]. It has been validated in a small study (n = 54) by van Gils, van Heugten [7] excluding survivors over 70 years and Nordström [8] that recommends combinig it with the Symbol Digit Modalities Test [37] to improve the sensitivity of the MoCA. However, we believe that further examination of the MoCA as a screening tool for the CA population is warranted, particularly regarding inter-rater reliability—especially for visuospatial/executive items that require subjective judgment. This is particularly important given that the average MoCA score in this population is very close to the cut-off [7, 8], making every point critical.

In our validation study, we compare the DANcSCA’s performance in correctly classifying individuals with that of the MoCA and the MoCA combined with the SDMT. Further investigation is needed but falls outside the scope of this development article.

Based on clinical experience from the 98 tests conducted in this study, we find it crucial to ensure that the presentation of screening results includes clear information on coping with any identified deficits and where to seek further help if needed. It is our belief that survivors should never be left alone with the knowledge that their scores have dropped below a specific cut-off without a plan for subsequent assessments and help from qualified personnel.

Our three-class approach offers significant benefits over a simple dichotomous test. It allows us to better differentiate between those with significant deficits, who should be referred for further assessment and rehabilitation without delay, and those with intermediate concerns, who may benefit from additional evaluation that considers their specific circumstances. This approach acknowledges that a screen cannot reliably distinguish between cognitive deficits resulting from emotional states versus brain injury. Additionally, it is important to consider an individual's cognitive reserves and the demands of their daily life when interpreting results [3].

### Strength and limitations

The development of the DANcSCA screening tool and procedure has been greatly strengthened by interdisciplinary collaboration, stakeholder involvement, and the valuable contributions of survivors and their relatives. This process ensured that the screen was grounded in practical needs and real-world applicability. However, in retrospect, the development process could have benefited from a more robust guiding framework from the outset. The clarity and structure provided by the GUIDED guidelines [14], which were published in April 2020, might have led to a shorter, more focused development process and more targeted data collection. Nonetheless, our development process naturally emerged from a genuine effort to find new and improved ways to assist CA survivors and their relatives.

Digital testing addresses the challenges of limited access to neuropsychologists and the shortcomings of coarse screening tools. However, this shift to digital testing has also introduced new challenges. These include navigating GDPR compliance, overcoming a lack of experience with digital tools, and facing scepticism from within the health professional community. A particular concern is that digital testing may not capture critical clinical impressions, such as a patient’s level of insight and awareness or the presence of neurological or psychiatric symptoms—elements that are essential in comprehensive neuropsychological testing [9]. While this concern is valid, it highlights the importance of considering further referral to a more comprehensive evaluation when needed. These challenges underscore the need for ongoing evaluation and refinement of the tool, particularly in ensuring that it complements rather than replaces the valuable insights provided by traditional neuropsychological assessments.

## Conclusion

The DANcSCA screening includes a digital tool, appropriate timing for administration, and a model for effectively communicating the results. The screening is feasible in clinical settings.

The next steps will be validating the screening tool to determine precise cutoff scores, further refining its usability, and addressing the challenges associated with digital testing in clinical practice. Additionally, interventions tailored to those with intermediate concerns need to be developed and tested, ensuring that the tool not only identifies deficits but also facilitates appropriate follow-up care.

## Supplementary Information

Supplementary Material 1.

## Data Availability

The data used and analysed during the current study are available from the corresponding author on reasonable request.

## References

[1] Ringreen K, Schoenau L, Lippert F. Yearly report on the Danish Cardiac Arrest Registry [årsrapport for Dansk Hjertestop Register](2020); October 16, 2021. 2022.

[2] Moulaert VRMP, Verbunt JA, van Heugten CM, Wade DT. Cognitive impairments in survivors of out-of-hospital cardiac arrest: A systematic review. Resuscitation. 2009;80(3):297–305. 19117659 10.1016/j.resuscitation.2008.10.034

[3] Zook N, Voss S, Nordström EB, Brett SJ, Jenkinson E, Shaw P, et al. Neurocognitive function following out-of-hospital cardiac arrest: A systematic review. Resuscitation. 2021. 10.1016/j.resuscitation.2021.10.005 34648921

[4] Nolan JP, Sandroni C, Böttiger BW, Cariou A, Cronberg T, Friberg H, et al. European Resuscitation Council and European Society of Intensive Care Medicine Guidelines 2021: Post-resuscitation care. Resuscitation. 2021;161:220–69. 33773827 10.1016/j.resuscitation.2021.02.012

[5] Steinbusch CVM, van Heugten CM, Rasquin SMC, Verbunt JA, Moulaert VRM. Cognitive impairments and subjective cognitive complaints after survival of cardiac arrest: A prospective longitudinal cohort study. Resuscitation. 2017;120:132–7. 28818523 10.1016/j.resuscitation.2017.08.007

[6] van Gils P, van Heugten C, Sep S, Moulaert V, Hofmeijer J, Verbunt J. A change of perspective? An explorative study on why patients may not subjectively report cognitive impairments after a cardiac arrest. Resuscitation. 2022;180:59–63. 36185035 10.1016/j.resuscitation.2022.09.008

[7] van Gils P, van Heugten C, Hofmeijer J, Keijzer H, Nutma S, Duits A. The Montreal Cognitive Assessment is a valid cognitive screening tool for cardiac arrest survivors. Resuscitation. 2022;172:130–6. 34958880 10.1016/j.resuscitation.2021.12.024

[8] Blennow Nordström E, Evald L, Mion M, Segerström M, Vestberg S, Ullén S, et al. Combined use of the Montreal Cognitive Assessment and Symbol Digit Modalities Test improves neurocognitive screening accuracy after cardiac arrest: A validation sub-study of the TTM2 trial. Resuscitation. 2024:110361. 10.1016/j.resuscitation.2024.110361 39147306

[9] Pedersen AD, Guldberg A, Riis J, Rune K, Hall N. Retningslinjer for neuropsykologiske undersøgelser og rehabilitering voksenområdet. https://neuropsykologiskpraksisdk/wp-content/uploads/4379133-retningslinjer-neurologiske-undersoegelser-2021-korr2-1pdf: Dansk Psykolog Forening; 2021.

[10] Podell K, Keller JM. Neuropsychological assessment. In: Coffey CE, Cummings JL, George MSW, D., editors. The American Psychiatric Publishing textbook of geriatric neuropsychiatry: American Psychiatric Publishing, Inc.; 2011. p. 121–45.

[11] Sherman E, Hrabok M. A compendium of neuropsychological tests: Fundamentals of neuropsychological assessment and test reviews for clinical practice: Oxford University Press; 2023.

[12] Moulaert VRM, van Heugten CM, Winkens B, Bakx WGM, de Krom MCFTM, Gorgels TPM, et al. Early neurologically-focused follow-up after cardiac arrest improves quality of life at one year: A randomised controlled trial. International Journal of Cardiology. 2015;193:8–16. 10.1016/j.ijcard.2015.04.229 26005166

[13] O’Cathain A, Croot L, Duncan E, Rousseau N, Sworn K, Turner KM, et al. Guidance on how to develop complex interventions to improve health and healthcare. BMJ Open. 2019;9(8): e029954. 31420394 10.1136/bmjopen-2019-029954 PMC6701588

[14] Duncan E, O’Cathain A, Rousseau N, Croot L, Sworn K, Turner KM, et al. Guidance for reporting intervention development studies in health research (GUIDED): an evidence-based consensus study. BMJ Open. 2020;10(4): e033516. 32273313 10.1136/bmjopen-2019-033516 PMC7245409

[15] Evald L, Brønnick K, Duez CHV, Grejs AM, Jeppesen AN, Søreide E, et al. Prolonged targeted temperature management reduces memory retrieval deficits six months post-cardiac arrest: A randomised controlled trial. Resuscitation. 2019;134:1–9. 30572070 10.1016/j.resuscitation.2018.12.002

[16] Lilja G, Nielsen N, Friberg H, Horn J, Kjaergaard J, Nilsson F, et al. Cognitive Function in Survivors of Out-of-Hospital Cardiac Arrest After Target Temperature Management at 33°C Versus 36°C. Circulation. 2015;131(15):1340–9. 25681466 10.1161/CIRCULATIONAHA.114.014414

[17] Nordström EB, Lilja G, Vestberg S, Nielsen N, Cronberg T. Neuropsychological outcome after cardiac arrest: Rationale and description of a sub-study of The Target Temperature Management 2 Trial. Resuscitation. 2018;130: e130.

[18] Tang LH, Joshi V, Egholm CL, Zwisler A-D. Are survivors of cardiac arrest provided with standard cardiac rehabilitation? – Results from a national survey of hospitals and municipalities in Denmark. Eur J Cardiovasc Nurs. 2020;20(2):115–23. 10.1177/1474515120946313 33849060

[19] Tang LH, Zwisler ADO, editors. Rehabilitering efter hjertestop uden for hospital–vi kan helt sikkert gøre det bedre!: Konsensus mellem hjertestopoverlevere, pårørende og fagprofessionelle. Cardiologisk Forum; 2019: Dansk Cardiologisk Selskab.

[20] Osterrieth PA. Le test de copie d'une figure complexe; contribution a l'etude de la perception et de la memoire. Archives de psychologie. 1944.

[21] Randolph C, Tierney MC, Mohr E, Chase TN. The Repeatable Battery for the Assessment of Neuropsychological Status (RBANS): preliminary clinical validity. J Clin Exp Neuropsychol. 1998;20(3):310–9. 10.1076/jcen.20.3.310.823 9845158

[22] Wechsler D. Wechsler adult intelligence scale. Arch Clin Neuropsychol. 1955.

[23] Brickenkamp R, Zillmer E. The D2 test of attention: Hogrefe & Huber; 1998.

[24] Reitan RM. The relation of the trail making test to organic brain damage. J Consult Psychol. 1955;19(5):393–4. 10.1037/h0044509 13263471

[25] Reitan RM. Validity of the trail making test as an indicator of organic brain damage. Percept Motor Skills. 1958;8:271–6.

[26] Meyers JE, Volkert K, Diep A. Sentence repetition test: updated norms and clinical utility. Appl Neuropsychol. 2000;7(3):154–9. 10.1207/S15324826AN0703_6 11125709

[27] Gronwall DMA. Paced auditory serial-addition task: a measure of recovery from concussion. Percept Mot Skills. 1977;44(2):367–73. 10.2466/pms.1977.44.2.367 866038

[28] Borkowski JG, Benton AL, Spreen O. Word fluency and brain damage. Neuropsychologia. 1967;5(2):135–40.

[29] Zec RF, Landreth ES, Fritz S, Grames E, Hasara A, Fraizer W, et al. A comparison of phonemic, semantic, and alternating word fluency in Parkinson's disease. Arch Clin Neuropsychol. 1999;14(3):255-64. 14590594

[30] Regard M, Strauss E, Knapp P. Children's production on verbal and non-verbal fluency tasks. Percept Mot Skills. 1982;55(3):839-44. 10.2466/pms.1982.55.3.839 7162920

[31] Parsey CM, Schmitter-Edgecombe M. Applications of Technology in Neuropsychological Assessment. Clin Neuropsychol. 2013;27(8):1328–61. 24041037 10.1080/13854046.2013.834971 PMC3869883

[32] Willer L, Pedersen PM, Forchhammer HB, Christensen H. Cognitive assessment at bedside for iPad: A preliminary validation of a novel cognitive test for stroke patients. Eur Stroke J. 2016;1(4):294–301. 31008291 10.1177/2396987316665233 PMC6301243

[33] Cambridge-Cognition. CANTAB®. 2019. p. Cognitive assessment software.

[34] Cambridge Cognition. Cambridge Neuropsychological Test Automated Battery 2024 [Available from: https://www.cambridgecognition.com/cantab/.

[35] Hoffmann TC, Glasziou PP, Boutron I, Milne R, Perera R, Moher D, et al. Better reporting of interventions: template for intervention description and replication (TIDieR) checklist and guide. BMJ. 2014;348: g1687. 24609605 10.1136/bmj.g1687

[36] Wagner MK, Berg SK, Hassager C, Borregaard B, Rasmussen TB, Ekholm O, et al. Cognitive impairment and psychopathology in sudden out-of-hospital cardiac arrest survivors: results from the REVIVAL cohort study. Resuscitation. 2023;192:109984. 10.1016/j.resuscitation.2023.109984 37797716

[37] Smith A. The Symbol Digit Modalities Test: A neutopsychologic test for economic screening of learning and other cerebral disorders. Learning Disorders. 1968;3(83):91.

