# Supplementary material for: Development of the DANish Cognitive Screen for Cardiac Arrest Survivors (DANcSCA)
Source: BMC Psychol. 2025 Apr 10;13:360. doi: 10.1186/s40359-025-02648-6 (PMC11984037; doi:10.1186/s40359-025-02648-6)
Supplement: Supplementary file 1 — Supplementary Material 1. [file 40359_2025_2648_MOESM1_ESM.docx]

**Appendix A**

**Digital cognitive test batteries**

The review, assessment, and decision were conducted by two psychologists in July 2019, with a follow-up in May 2021. Through searching the Internet and scientific literature, we identified the following list of digital test batteries capable of assessing the required cognitive functions. The assessment of the digital test batteries was based on the following criteria.

- Available in Danish
- Well used and studied.
- User-friendliness – front and backend
- Available for clinical use
- No need for certified personal
- Well-established organisation
- Relevant tests for the CA population

|  | Danish | Used & studied | User-friendliness | Clinical use | Certified personal | Well-established |
| --- | --- | --- | --- | --- | --- | --- |
| Automated Neuropsychological Assessment Metrics | 🗷 | 🗹 | 🗹 | 🗹 | ? | 🗹 |
| Cambridge Neuropsychological Test Automated Battery, CANTAB | 🗹 | 🗹 | 🗹 | 🗹 | 🗹 | 🗹 |
| CAMCI®-Research | 🗷 | 🗹 | 🗹 | 🗷 | ? | 🗹 |
| Cognitive Assessment at Bedside for iPad (CABPad) | 🗹 | 🗷 | ? | 🗹 | 🗹 | 🗷 |
| Cognitive Function Scanner | 🗹 | 🗷 | ? | 🗹 | ? | 🗷 |
| Cogstate C3 | ? | 🗹 | 🗹 | 🗹 | ? | 🗹 |
| NIH Toolbox | 🗷 | 🗹 | 🗹 | 🗹 | ? | 🗹 |
| OCS-Plus | 🗷 | 🗷 | 🗹 | 🗹 | ? | 🗷 |
| Wechsler Q-Interactive | 🗹 | 🗷 | 🗹 | 🗹 | ? | 🗷 |

We decided to compile the digital screening tool using subtests from CANTAB since the assessment from the dedicated research group was that CANTAB was the system with the greatest potential to be administered without assistance from health personnel. Furthermore, CANTAB was the most user-friendly and comprehensive test battery. It was the battery that most easily could overcome the potential pitfalls of digital tools as outlined by Germine et al. (Germine et al., 2019)

CANTAB had, at the time, more than 2.200 citations (Cambridge Cognition, 2024). CANTAB’s ability to discriminate between healthy and clinical populations was supported, although not specific enough to discern one cognitive function from another (Lenehan et al., 2016). For further comparisons of CANTAB with traditional neuropsychological tests, see (Lenehan et al., 2016; Schulz-Heik et al., 2020; Smith et al., 2013).

References

Cambridge Cognition. (2024). *Cambridge Neuropsychological Test Automated Battery*. <https://www.cambridgecognition.com/cantab/>

Germine, L., Reinecke, K., & Chaytor, N. S. (2019). Digital neuropsychology: Challenges and opportunities at the intersection of science and software. *The Clinical Neuropsychologist*, *33*(2), 271-286. <https://doi.org/10.1080/13854046.2018.1535662>

Lenehan, M. E., Summers, M. J., Saunders, N. L., Summers, J. J., & Vickers, J. C. (2016). Does the Cambridge Automated Neuropsychological Test Battery (CANTAB) Distinguish Between Cognitive Domains in Healthy Older Adults? *Assessment*, *23*(2), 163-172. <https://doi.org/10.1177/1073191115581474>

Schulz-Heik, R. J., Fahimi, A., Durazzo, T. C., Friedman, M., & Bayley, P. J. (2020). Evaluation of adding the CANTAB computerized neuropsychological assessment battery to a traditional battery in a tertiary care center for veterans. *Applied Neuropsychology: Adult*, *27*(3), 256-266. <https://doi.org/10.1080/23279095.2018.1534735>

Smith, P. J., Need, A. C., Cirulli, E. T., Chiba-Falek, O., & Attix, D. K. (2013). A comparison of the Cambridge Automated Neuropsychological Test Battery (CANTAB) with “traditional” neuropsychological testing instruments. *Journal of Clinical and Experimental Neuropsychology*, *35*, 319-328. <https://doi.org/10.1080/13803395.2013.771618>
